# The genome sequence of the lesser worm flesh fly, *Sarcophaga* ( *Sarcophaga*) *subvicina* (Baranov, 1937)

**DOI:** 10.12688/wellcomeopenres.18717.1

**Published:** 2023-02-08

**Authors:** Steven Falk, University of Oxford and Wytham Woods Genome Acquisition Lab, Darwin Tree of Life Barcoding collective, Wellcome Sanger Institute Tree of Life programme, Wellcome Sanger Institute Scientific Operations: DNA Pipelines collective, Tree of Life Core Informatics collective, John F. Mulley, Darwin Tree of Life Consortium

**Affiliations:** 1 independent Researcher, Kenilworth, Warwickshire, UK; 2 School of Natural Sciences, Bangor University, Bangor, Wales, UK

**Keywords:** Sarcophaga subvicina, lesser worm flesh fly, genome sequence, chromosomal, Diptera

## Abstract

We present a genome assembly from an individual male *Sarcophaga subvicina *(the lesser worm flesh fly; Arthropoda; Insecta; Diptera; Sarcophagidae). The genome sequence is 71 megabases in span. Most of the assembly (95.91%) is scaffolded into six chromosomal pseudomolecules, with the X sex chromosome assembled. The mitochondrial genome has also been assembled and is 16.7 kilobases in length. Gene annotation of this assembly on Ensembl identified 16,793 protein coding genes.

## Species taxonomy

Eukaryota; Metazoa; Ecdysozoa; Arthropoda; Hexapoda; Insecta; Pterygota; Neoptera; Endopterygota; Diptera; Brachycera; Muscomorpha; Oestroidea; Sarcophagidae; Sarcophaga; *Sarcophaga*; *Sarcophaga subvicina* (Baranov, 1937) (NCBI txid:236850).

## Background

*Sarcophaga subvicina* (Diptera: Sarcophagidae) is a relatively large (up to 8–15 mm ( van Emden, 1954)) flesh fly with a Nearartic and Palearctic distribution ( Pape, 1996). *S. subvicina* show the characteristic patterning of the Sarocophaga genus, with an overall blackish/greyish colouration, a checked abdomen, three longitudinal stripes on the thorax, and large red/orange eyes, and so can be difficult to separate from other members of the genus without examination of male genitalia or DNA barcoding ( Jordaens *et al.*, 2013; Szpila *et al.*, 2015). *Sarcophaga* is a large genus, and the nearly 900 species contained within it are classified into 169 subgenera ( Buenaventura *et al.*, 2017), with *S. subvicina* placed in the *Sarcophaga* subgenus along with over 20 other species ( Pape, 1996). The relative species-richness of this subgenus stands in stark contrast to the majority of Sarcophagid subgenera, which are monotypic. The *Sarcophaga* subgenus contains three of the roughly 65 currently recognised UK Sarcophagid species ( *S. carnaria*, *S. variegata*, and *S. subvicina*), in what is often termed the “carnaria group”.

*Sarcophaga subvicina* is found across the UK, with a range that extends to the north of Scotland, and is most abundant between May and September (see: https://species.nbnatlas.org/species/NBNSYS0000030329). It has been reported as favouring open (urban/grassland) habitats ( Fremdt & Amendt, 2014; Hwang & Turner, 2005), and adults have been attracted to large carcasses ( Szpila *et al.*, 2015). Larvae have been reported only from small mammal carcasses, and reared in captivity on meat and dead slugs ( Blackith & Blackith, 1994; Pape, 1987), but this species seems to more likely represent an earthworm specialist. All Sarcophagids examined to date have a diploid chromosome number of 12, with an XY sex determination system and males the heterogametic sex ( Srivastava & Gaur, 2015).

The genome of the lesser worm flesh fly *S. subvicina* was sequenced as part of the Darwin Tree of Life Project, a collaborative effort to sequence all named eukaryotic species in the Atlantic Archipelago of Britain and Ireland. Here we present a chromosomally complete genome sequence for *S. subvicina* based on an individual male specimen from Wytham Woods, Berkshire.

### Genome sequence report

**Figure 1.**
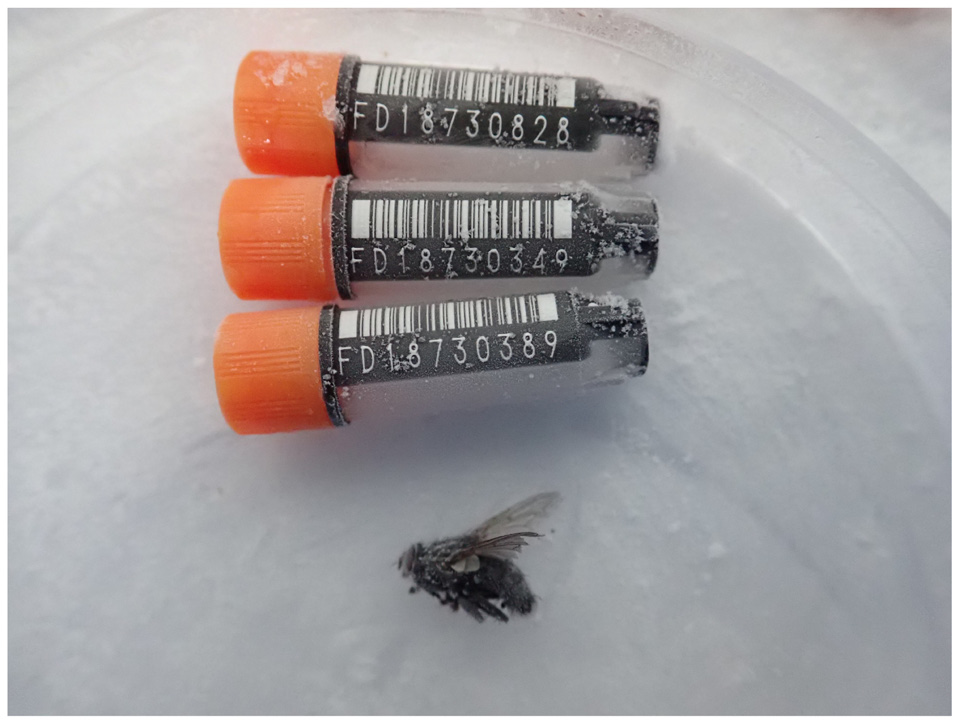
Image of the *Sarcophaga subvicina* (idSarSubv1) specimen used for genome sequencing.

The genome was sequenced from one male *S. subvicina* specimen collected in Wytham Woods, Berkshire ( Figure 1). A total of 65-fold coverage in Pacific Biosciences single-molecule HiFi long reads and 51-fold coverage in 10X Genomics read clouds were generated. Primary assembly contigs were scaffolded with chromosome conformation Hi-C data. Manual assembly curation corrected 95 missing/misjoins and removed four haplotypic duplications, reducing the assembly length by 0.57% and the scaffold number by 16.97%, and increasing the scaffold N50 by 4.73%.

**Figure 2.**
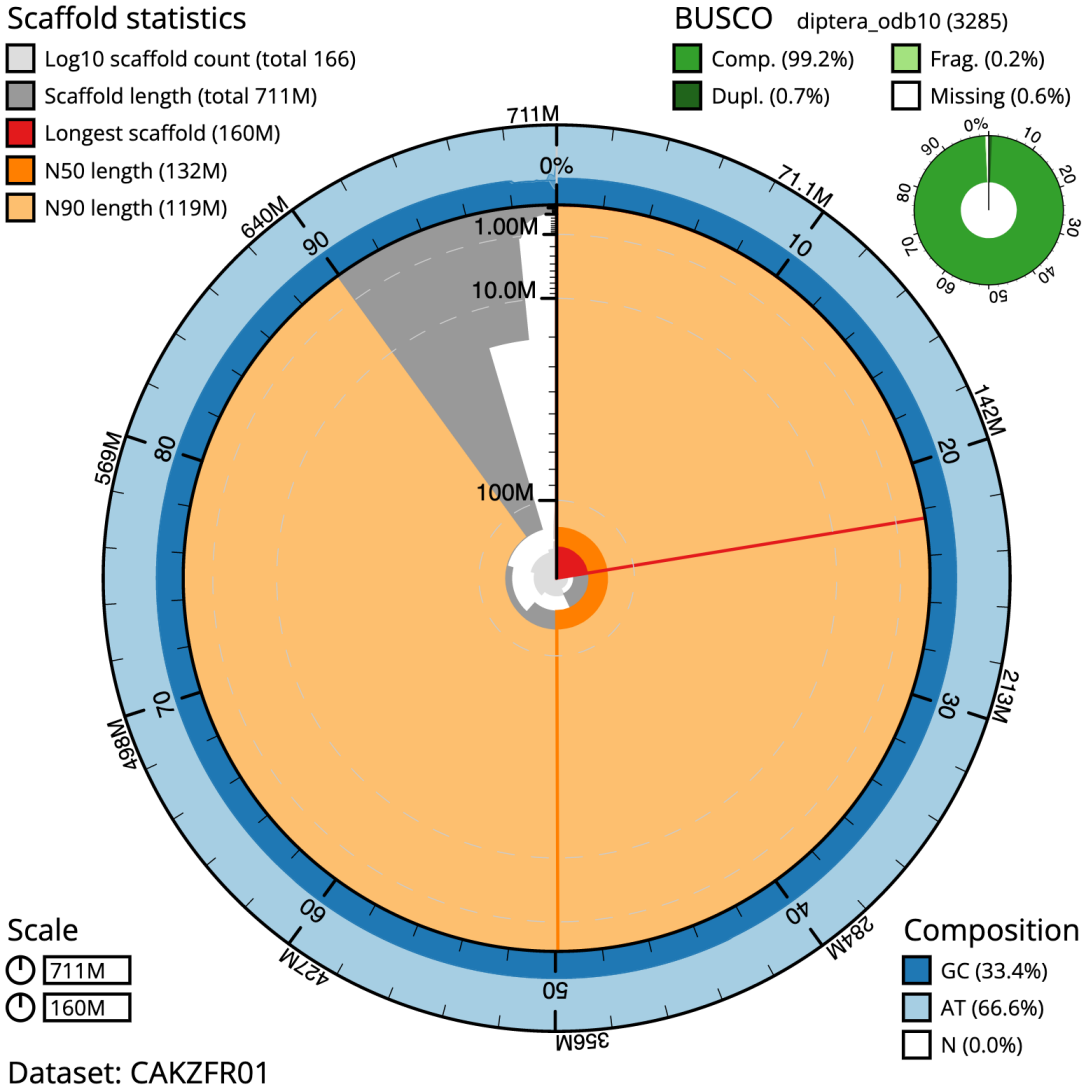
Genome assembly of *Sarcophaga subvicina*, idSarSubv1.1: metrics. The BlobToolKit Snailplot shows N50 metrics and BUSCO gene completeness. The main plot is divided into 1,000 size-ordered bins around the circumference with each bin representing 0.1% of the 711,151,016 bp assembly. The distribution of chromosome lengths is shown in dark grey with the plot radius scaled to the longest chromosome present in the assembly (159,501,612bp, shown in red). Orange and pale-orange arcs show the N50 and N90 chromosome lengths (132,242,496 and 118,606,681bp), respectively. The pale grey spiral shows the cumulative chromosome count on a log scale with white scale lines showing successive orders of magnitude. The blue and pale-blue area around the outside of the plot shows the distribution of GC, AT and N percentages in the same bins as the inner plot. A summary of complete, fragmented, duplicated and missing BUSCO genes in the diptera_odb10 set is shown in the top right. An interactive version of this figure is available at https://blobtoolkit.genomehubs.org/view/idSarSubv1.1/dataset/CAKZFR01/snail.

The final assembly has a total length of 714 Mb in 274 sequence scaffolds with a scaffold N50 of 123 Mb ( Table 1). Most (95.91%) of the assembly sequence was assigned to six chromosomal-level scaffolds, representing 5 autosomes and the X sex chromosome ( Figure 2– Figure 5; Table 2). Chromosome-scale scaffolds confirmed by the Hi-C data are named in order of size. This is a male specimen with known XY sex determination system, however we have been unable to identify Y sequences. The X chromosome is assembled from scaffolds of undetermined order and orientation. The assembly has a BUSCO 5.3.2 ( Manni *et al.*, 2021) completeness of 99.2% (single 98.5%, duplicated 0.7%), using the diptera_odb10 reference set ( *n* = 3,285). While not fully phased, the assembly deposited is of one haplotype. Contigs corresponding to the second haplotype have also been deposited.

**Table 1.** Genome data for idSarSubv1.1.

Project accession data	
Assembly identifier	idSarSubv1.1
Species	*Sarcophaga subvicina*
Specimen	idSarSubv1
NCBI taxonomy ID	236850
BioProject	PRJEB51465
BioSample ID	SAMEA7746447
Isolate information	male, thorax tissue (genomic DNA), head tissue (Hi-C)
Assembly metrics *	
Base pair QV	52.9 (Benchmark: ≥50)
*k*-mer completeness	99.99% (Benchmark: ≥95%)
BUSCO **	C:99.2%[S:98.5%,D:0.7%],F:0.2%,M:0.6%,n:3285 (Benchmark: C ≥ 95%)
Percentage of assembly mapped to chromosomes	95.91% (Benchmark: ≥95%)
Sex chromosomes	X chromosome identified (Benchmark: localised homologous pairs)
Organelles	Mitochondrion genome assembled (Benchmark: complete single alleles)
Raw data accessions	
PacificBiosciences SEQUEL II	ERR9284049, ERR9284050
10X Genomics Illumina	ERR9248453–ERR9248456
Hi-C Illumina	ERR9248452
Genome assembly	
Assembly accession	GCA_936449025.1
Accession of alternate haplotype	GCA_936440885.1
Span (Mb)	714.2
Number of contigs	445
Contig N50 length (Mb)	102.9
Number of scaffolds	274
Scaffold N50 length (Mb)	122.7
Longest scaffold (Mb)	159.5
Genome annotation	
Number of protein-coding genes	16,793

* Assembly metric benchmarks are adapted from column VGP-2020 of “Table 1: Proposed standards and metrics for defining genome assembly quality” from ( Rhie *et al.*, 2021). ** BUSCO scores based on the diptera_odb10 BUSCO set using v5.3.2. C = complete [S = single copy, D = duplicated], F = fragmented, M = missing, n = number of orthologues in comparison. A full set of BUSCO scores is available at https://blobtoolkit.genomehubs.org/view/idSarSubv1.1/dataset/CAKZFR01/busco.

**Figure 3.**
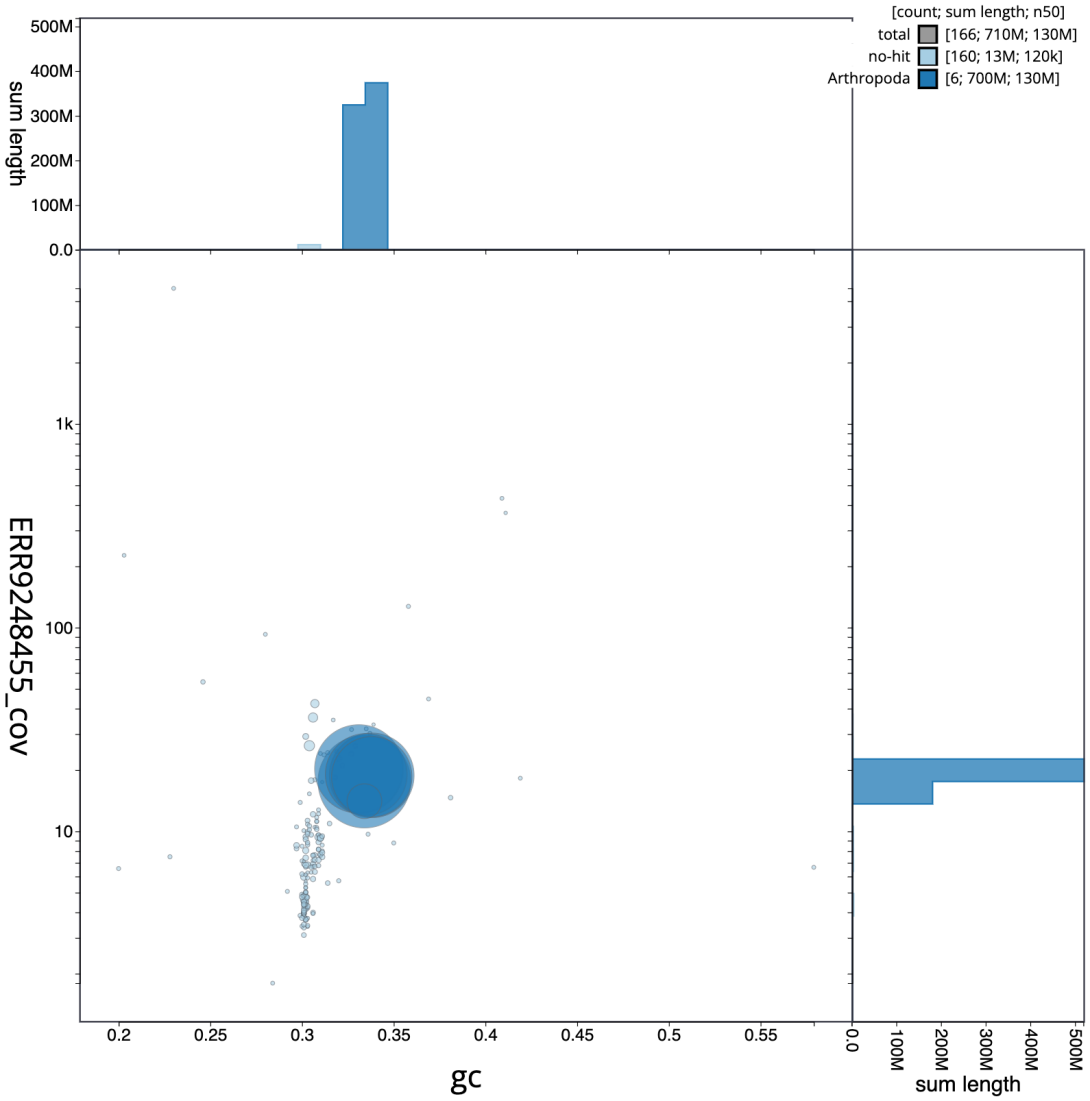
Genome assembly of *Sarcophaga subvicina*, idSarSubv1.1: GC coverage. BlobToolKit GC-coverage plot. Chromosomes are coloured by phylum. Circles are sized in proportion to chromosome length. Histograms show the distribution of chromosome length sum along each axis. An interactive version of this figure is available at https://blobtoolkit.genomehubs.org/view/idSarSubv1.1/dataset/CAKZFR01/blob.

**Figure 4.**
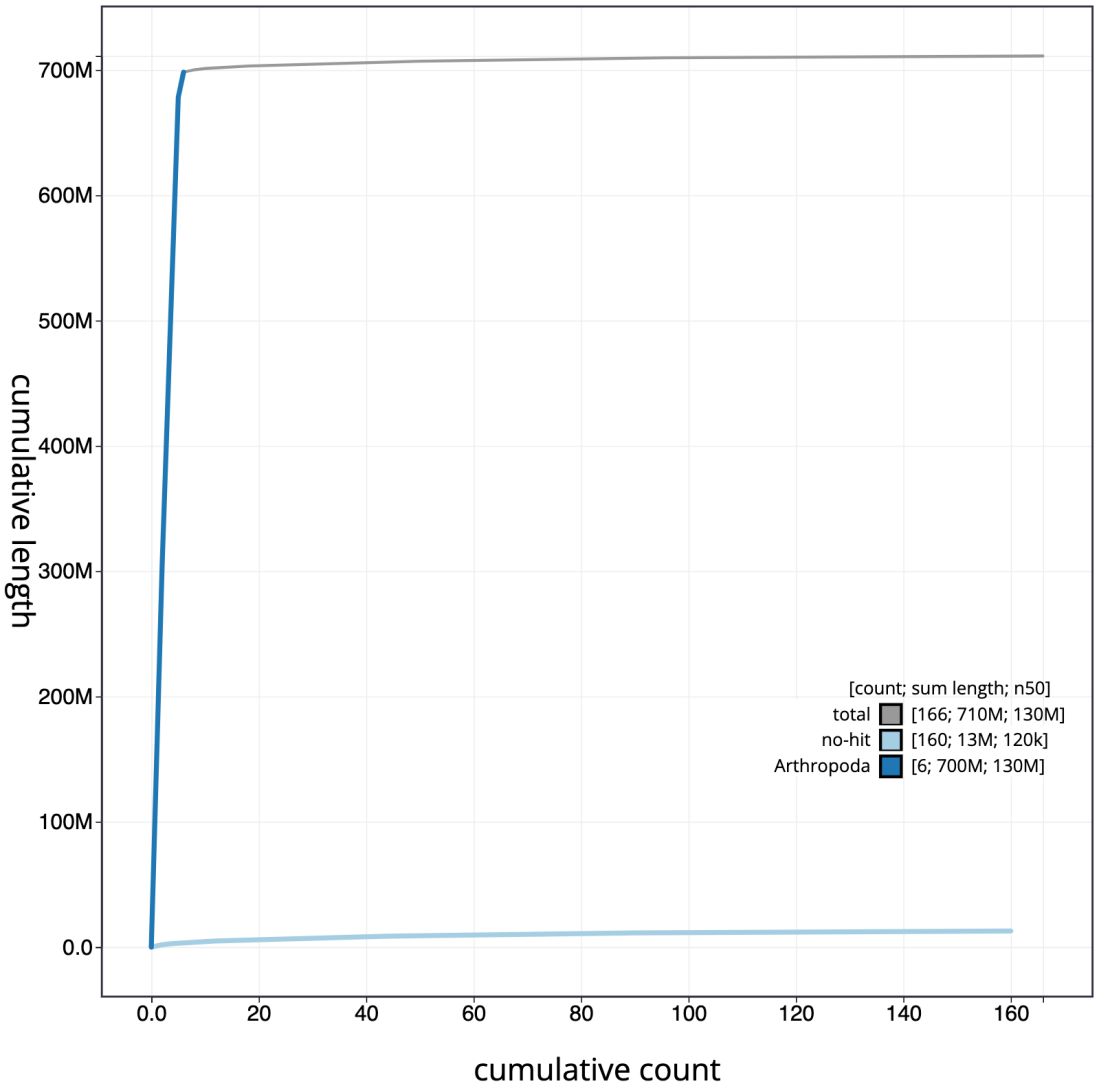
Genome assembly of *Sarcophaga subvicina*, idSarSubv1.1: cumulative sequence. BlobToolKit cumulative sequence plot. The grey line shows cumulative length for all chromosomes. Coloured lines show cumulative lengths of chromosomes assigned to each phylum using the buscogenes taxrule. An interactive version of this figure is available at https://blobtoolkit.genomehubs.org/view/idSarSubv1.1/dataset/CAKZFR01/cumulative.

**Figure 5.**
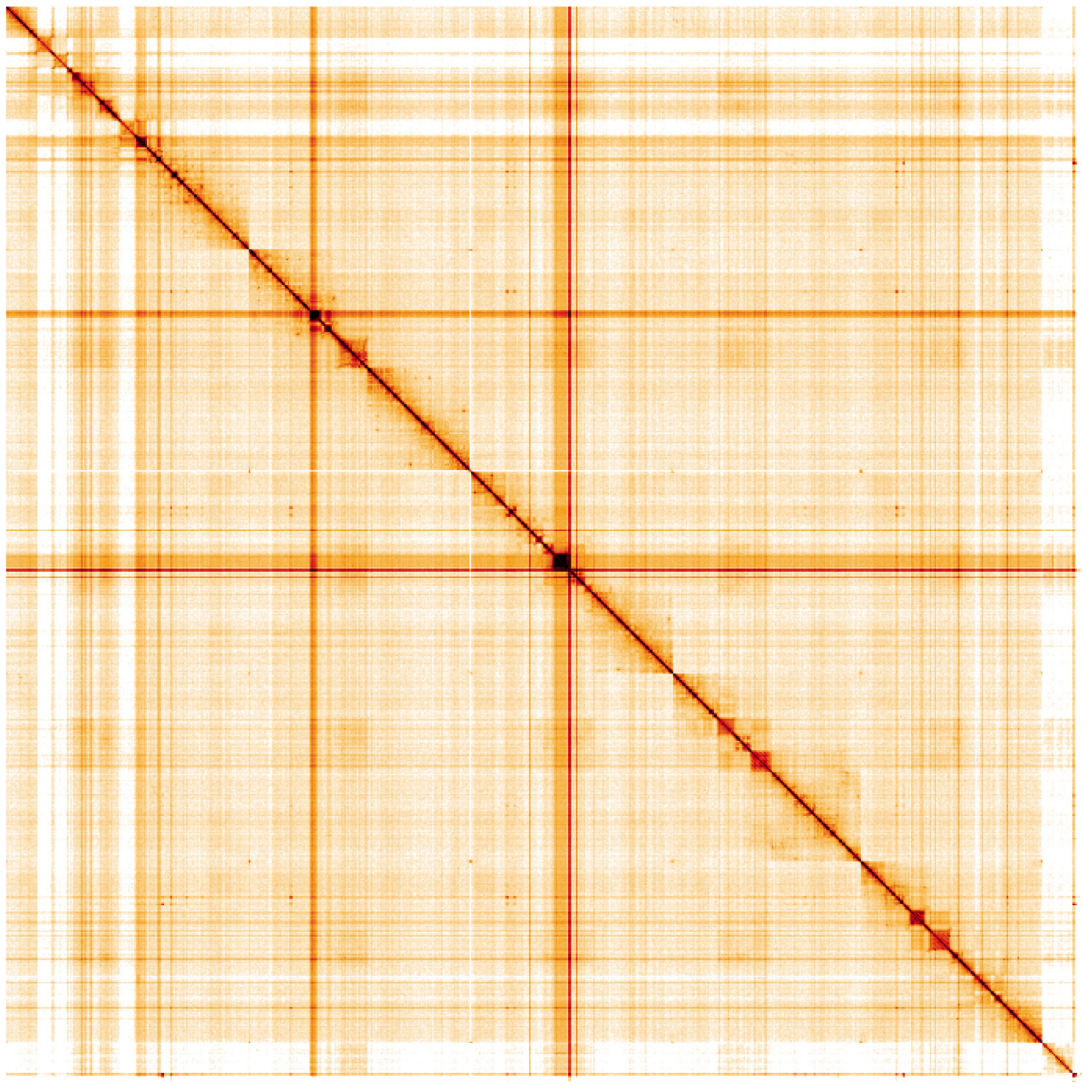
Genome assembly of *Sarcophaga subvicina*, idSarSubv1.1: Hi-C contact map. Hi-C contact map of the idSarSubv1.1 assembly, visualised using HiGlass. Chromosomes are shown in order of size from left to right and top to bottom. An interactive version of this figure may be viewed at https://genome-note-higlass.tol.sanger.ac.uk/l/?d=EyLpsE98QiGBosMm1WaaPw.

**Table 2.** Chromosomal pseudomolecules in the genome assembly of *Sarcophaga subvicina*, idSarSubv1.

INSDC accession	Chromosome	Size (Mb)	GC%
OW388080.1	1	159.5	33.4
OW388081.1	2	144.87	33.1
OW388082.1	3	132.24	33.8
OW388083.1	4	122.97	33.5
OW388084.1	5	118.61	33.8
OW388085.1	X	20.1	33.4
OW388086.1	MT	0.02	23

### Genome annotation report

The idSarSubv1.1 genome was annotated using the Ensembl rapid annotation pipeline ( Table 1; https://rapid.ensembl.org/Sarcophaga_subvicina_GCA_936449025.1/). The resulting annotation includes 39,250 transcribed mRNAs from 16,793 protein-coding and 11,903 non-coding genes.

## Methods

### Sample acquisition and nucleic acid extraction

A male *S. subvicina* (idSarSubv1) was collected and identified by Steven Falk (independent researcher). The specimen was collected using a net in Wytham Woods, Berkshire (latitude 51.766, longitude –1.309) and snap-frozen on dry ice.

DNA was extracted at the Tree of Life laboratory, Wellcome Sanger Institute. The idSarSubv1 sample was weighed and dissected on dry ice with head tissue set aside for Hi-C sequencing. Thorax tissue was *[[if powermasher used:* disrupted using a Nippi Powermasher fitted with a BioMasher pestle; *else:* cryogenically disrupted to a fine powder using a Covaris cryoPREP Automated Dry Pulveriser, receiving multiple impacts *]]*. High molecular weight (HMW) DNA was extracted using the Qiagen MagAttract HMW DNA extraction kit. Low molecular weight DNA was removed from a 20 ng aliquot of extracted DNA using 0.8X AMpure XP purification kit prior to 10X Chromium sequencing; a minimum of 50 ng DNA was submitted for 10X sequencing. HMW DNA was sheared into an average fragment size of 12–20 kb in a Megaruptor 3 system with speed setting 30. Sheared DNA was purified by solid-phase reversible immobilisation using AMPure PB beads with a 1.8X ratio of beads to sample to remove the shorter fragments and concentrate the DNA sample. The concentration of the sheared and purified DNA was assessed using a Nanodrop spectrophotometer and Qubit Fluorometer and Qubit dsDNA High Sensitivity Assay kit. Fragment size distribution was evaluated by running the sample on the FemtoPulse system.

### Sequencing

Pacific Biosciences HiFi circular consensus and 10X Genomics read cloud DNA sequencing libraries were constructed according to the manufacturers’ instructions. DNA sequencing was performed by the Scientific Operations core at the WSI on Pacific Biosciences SEQUEL II (HiFi) and Illumina NovaSeq 6000 (10X) instruments. Hi-C data were also generated from head tissue of idSarSubv1 using the Arima v2 kit and sequenced on the Illumina NovaSeq 6000 instrument.

### Genome assembly

Assembly was carried out with Hifiasm ( Cheng *et al.*, 2021) and haplotypic duplication was identified and removed with purge_dups ( Guan *et al.*, 2020). One round of polishing was performed by aligning 10X Genomics read data to the assembly with Long Ranger ALIGN, calling variants with freebayes ( Garrison & Marth, 2012). The assembly was then scaffolded with Hi-C data ( Rao *et al.*, 2014) using YaHS ( Zhou *et al.*, 2022). The assembly was checked for contamination as described previously ( Howe *et al.*, 2021). Manual curation was performed using HiGlass ( Kerpedjiev *et al.*, 2018) and Pretext ( Harry, 2022). The mitochondrial genome was assembled using MitoHiFi ( Uliano-Silva *et al.*, 2021), which performed annotation using MitoFinder ( Allio *et al.*, 2020). The genome was analysed and BUSCO scores generated within the BlobToolKit environment ( Challis *et al.*, 2020). Table 3 contains a list of all software tool versions used, where appropriate.

**Table 3.** Software tools and versions used.

Software tool	Version	Source
BlobToolKit	3.4.0	Challis *et al*., 2020
freebayes	1.3.1-17- gaa2ace8	Garrison & Marth, 2012
Hifiasm	0.15.3	Cheng *et al*., 2021
HiGlass	1.11.6	Kerpedjiev *et al*., 2018
Long Ranger ALIGN	2.2.2	https://support.10xgenomics.com/genome-exome/software/pipelines/latest/advanced/other-pipelines
MitoHiFi	2.0	Uliano-Silva *et al*., 2021
PretextView	0.2.x	https://github.com/wtsi-hpag/PretextView
purge_dups	1.2.3	Guan *et al*., 2020
YaHS	yahs-1.1.91eebc2	Zhou *et al*., 2022

### Genome annotation

The Ensembl gene annotation system ( Aken *et al.*, 2016) was used to generate annotation for the *S. subvicina* assembly (GCA_936449025.1). Annotation was created primarily through alignment of transcriptomic data to the genome, with gap filling via protein to-genome alignments of a select set of proteins from UniProt ( UniProt Consortium, 2019).

### Ethics/compliance issues

The materials that have contributed to this genome note have been supplied by a Darwin Tree of Life Partner. The submission of materials by a Darwin Tree of Life Partner is subject to the Darwin Tree of Life Project Sampling Code of Practice. By agreeing with and signing up to the Sampling Code of Practice, the Darwin Tree of Life Partner agrees they will meet the legal and ethical requirements and standards set out within this document in respect of all samples acquired for, and supplied to, the Darwin Tree of Life Project. Each transfer of samples is further undertaken according to a Research Collaboration Agreement or Material Transfer Agreement entered into by the Darwin Tree of Life Partner, Genome Research Limited (operating as the Wellcome Sanger Institute), and in some circumstances other Darwin Tree of Life collaborators.

## Data Availability

European Nucleotide Archive: *Sarcophaga subvicina*. Accession number PRJEB51465; https://identifiers.org/ena.embl/PRJEB51465 ( Wellcome Sanger Institute, 2022). The genome sequence is released openly for reuse. The *Sarcophaga subvicina* genome sequencing initiative is part of the Darwin Tree of Life (DToL) project. All raw sequence data and the assembly have been deposited in INSDC databases. Raw data and assembly accession identifiers are reported in Table 1.

## Author information

Members of the University of Oxford and Wytham Woods Genome Acquisition Lab are listed here: https://doi.org/10.5281/zenodo.4789928.

Members of the Darwin Tree of Life Barcoding collective are listed here: https://doi.org/10.5281/zenodo.4893703.

Members of the Wellcome Sanger Institute Tree of Life programme are listed here: https://doi.org/10.5281/zenodo.4783585.

Members of Wellcome Sanger Institute Scientific Operations: DNA Pipelines collective are listed here: https://doi.org/10.5281/zenodo.4790455.

Members of the Tree of Life Core Informatics collective are listed here: https://doi.org/10.5281/zenodo.5013541.

Members of the Darwin Tree of Life Consortium are listed here: https://doi.org/10.5281/zenodo.4783558.
