# Supplementary material for: The genome sequence of the lesser worm flesh fly, Sarcophaga ( Sarcophaga) subvicina Rohdendorf, 1937
Source: Wellcome Open Res. 2024 Sep 4;8:65. Originally published 2023 Feb 8. [Version 2] doi: 10.12688/wellcomeopenres.18717.2 (PMC10435921; doi:10.12688/wellcomeopenres.18717.2)
Supplement: Peer review history

# Reviewer report: Reviewer response for version 2

**DOI:** 10.21956/wellcomeopenres.24707.r109727

**Published:** 2024-11-20

**Authors:** Ruiqi Li

**Affiliations:** 1 University of Colorado Boulder, Boulder, Colorado, USA

The genome of *Sarcophaga* *subvicina* is high-quality. I only have a few minor comments:

 1. It would be great if the authors could provide a paragraph describing what biological questions this genome could help us answer. See some examples here: Li et al 2024a,b

 2. Add a scale to Fig. 1?

Are sufficient details of methods and materials provided to allow replication by others?

Yes

Is the rationale for creating the dataset(s) clearly described?

Yes

Are the datasets clearly presented in a useable and accessible format?

Yes

Are the protocols appropriate and is the work technically sound?

Yes

Reviewer Expertise:

Genomics

I confirm that I have read this submission and believe that I have an appropriate level of expertise to confirm that it is of an acceptable scientific standard.

## References

[1] : The genome sequence of the giant clam, Tridacna gigas (Linnaeus, 1758). *Wellcome Open Res* .2024;9: 10.12688/wellcomeopenres.21136.1 145 10.12688/wellcomeopenres.21136.1 38800516 PMC11116938

[2] : The genome sequence of the giant clam, Tridacna crocea (Lamarck, 1819). *Wellcome Open Research* .2023;8: 10.12688/wellcomeopenres.20580.1 10.12688/wellcomeopenres.20580.1

# Reviewer report: Reviewer response for version 2

**DOI:** 10.21956/wellcomeopenres.24707.r109725

**Published:** 2024-11-05

**Authors:** Jason Charamis

**Affiliations:** 1 Foundation for Research and Technology - Hellas, Irákleion, Greece

This study presents the genome assembly and annotation of *Sarcophaga subvicina*, a representative of lesser worm flesh flies. The work is technically sound and the generated genome assembly seems of great quality.

 I have only a few requests before acceptance for indexing:

 1. "The genome sequence is 71 megabases in span." : Typo should be corrected in the following sentence from Abstract - assembly size is 714 Mb

 2. "Annotation was created primarily through alignment of transcriptomic data to the genome": the transcriptomic evidence used as hints for gene prediction are not mentioned, while new RNA sequencing data are not produced within the frame of this study

 3. The number of predicted protein-coding genes is within the typical range for Diptera. To my view, what is currently missing is a BUSCO assessment of the ENSEMBL-produced gene annotation and a comparison with other reference dipterans, such as Drosophila melanogaster and/or other related species.

Are sufficient details of methods and materials provided to allow replication by others?

Yes

Is the rationale for creating the dataset(s) clearly described?

Yes

Are the datasets clearly presented in a useable and accessible format?

Yes

Are the protocols appropriate and is the work technically sound?

Yes

Reviewer Expertise:

Arthropod Comparative Genomics

I confirm that I have read this submission and believe that I have an appropriate level of expertise to confirm that it is of an acceptable scientific standard.

# Reviewer report: Reviewer response for version 2

**DOI:** 10.21956/wellcomeopenres.24707.r108255

**Published:** 2024-11-04

**Authors:** Jaakko Pohjoismäki

**Affiliations:** 1 University of Eastern Finland, Joensuu, Finland

The data note by Steven Falk and others with the DToL consortium presents the reference-quality genome assembly of *Sarcophaga subvicina*. The assembly meets the EBP standards and relevant data related to its assembly, annotation and QC, as well as sample metadata are provided under links.

 The note has been already reviewed once and the authors have made amendments accordingly. I have only few minor comments.

**Background**

 1) Twice )) in the first reference.

 2) Besides its *COI*-barcode, *Sarcophaga subvicina* can be reliably distinguished from the otherwise similar *carnaria-*group species only by the morphology of male genitalia. While I do not doubt the identification, especially as the barcoding was done, I would be curious to know how the snap-frozen specimen was handled, as the genitalia would need to be examined somehow. This is relevant because one needs to usually examine dozens of more common species (such as *subvicina*) to find one of the less abundant ones. I'm fine if the identification was made post-hoc, just wanted to know if the authors had found some solution for the trade-off between the requirement for time consuming morphological determination of unfrozen specimens, which can be manipulated to show the necessary details vs snap freezing them for the genome work.

 3) Species in the *carnaria*-group should all be obligate predators/parasitoids of earthworms, records from carrions are likely misidentifications. While this is mentioned, it is not really referenced, but rather the focus is on trivia (attracted to large carcasses, breeding in captivity).

**Methods**

 A point raised by an earlier review: It is mentioned that RNA-seq data was used to aid the annotation, but this has not been explained in the methods. I see from the sample metadata that head, thorax and abdomen were treated as separate samples. Methods state that the head was used for Hi-C and the thorax for the PacBio sequencing. I assume that the abdomen was then used for the RNA-seq?

 As a note to the previous reviews, I do not find it necessary to find justifications for sequencing a genome for a species nor detailed analysis of the genome structure etc. Production of genome assemblies will provide an excellent resource for any such future work and these genome consortia can focus only on their production and has value as such.

Are sufficient details of methods and materials provided to allow replication by others?

Yes

Is the rationale for creating the dataset(s) clearly described?

Yes

Are the datasets clearly presented in a useable and accessible format?

Yes

Are the protocols appropriate and is the work technically sound?

Yes

Reviewer Expertise:

Molecular biology; biodiversity genetics; taxonomy

I confirm that I have read this submission and believe that I have an appropriate level of expertise to confirm that it is of an acceptable scientific standard, however I have significant reservations, as outlined above.

# Reviewer report: Reviewer response for version 2

**DOI:** 10.21956/wellcomeopenres.24707.r97290

**Published:** 2024-09-19

**Authors:** Andrzej Grzywacz

**Affiliations:** 1 Nicolaus Copernicus University, Toruń, Poland

The authors appropriately addressed the issues raised during the previous revision round.

Are sufficient details of methods and materials provided to allow replication by others?

Partly

Is the rationale for creating the dataset(s) clearly described?

No

Are the datasets clearly presented in a useable and accessible format?

Yes

Are the protocols appropriate and is the work technically sound?

Yes

Reviewer Expertise:

Systematics, entomology, genomics

I confirm that I have read this submission and believe that I have an appropriate level of expertise to confirm that it is of an acceptable scientific standard.

# Reviewer report: Reviewer response for version 1

**DOI:** 10.21956/wellcomeopenres.20755.r63100

**Published:** 2023-08-17

**Authors:** Andrzej Grzywacz

**Affiliations:** 1 Nicolaus Copernicus University, Toruń, Poland

The report describes the results of extraction, sequencing and assembly of genome of *Sarcophaga subvicina*. The protocols of molecular work are well described. Some remarks consider other aspects of this work.

 An error is present in the title of this report. *Sarcophaga *( *Sarcophaga*) *subvicina *has been described by Rohdendorf, not by Baranov. The correct title should include:

*"Sarcophaga* ( *Sarcophaga*) *subvicina* Rohdendorf, 1937".

 According to the catalogue of Pape (1996) it is a species of Palaearctic distribution. Please delete "Neararctic" [sic!].

 Please correct cases where taxon names are not italicised.

 Please change "Sarcophagid" to "sarcophagid".

 Please provide some potential examples of studies where generated dataset can be employed.

 I recommend adding more information concerning specimen identification. In particular, a reference to a taxonomic key used for species delimitation, i.e., latest species-level taxonomic literature that contains the currently accepted species concept (Meier 2017).

 In the Methods section, please check whether a sentence "Thorax tissue was *[[if powermasher used:* disrupted using a Nippi Powermasher fitted with a BioMasher pestle; *else:* cryogenically disrupted to a fine powder using a Covaris cryoPREP Automated Dry Pulveriser, receiving multiple impacts *]]*." is correct.

Are sufficient details of methods and materials provided to allow replication by others?

Partly

Is the rationale for creating the dataset(s) clearly described?

No

Are the datasets clearly presented in a useable and accessible format?

Yes

Are the protocols appropriate and is the work technically sound?

Yes

Reviewer Expertise:

Systematics, entomology, genomics

I confirm that I have read this submission and believe that I have an appropriate level of expertise to confirm that it is of an acceptable scientific standard, however I have significant reservations, as outlined above.

## References

[1] : Catalogue of the Sarcophagidae of the world (Insecta: Diptera). *Associated Publishers* .1996;

[2] : Citation of taxonomic publications: the why, when, what and what not. *Systematic Entomology* .2017;42(2) : 10.1111/syen.12215 301-304 10.1111/syen.12215

# Response

**Published:** 2024-05-09

**Authors:** Tree of Life Team Sanger

**Affiliations:** Wellcome Trust Sanger Institute, UK

Thank you for the helpful comments on this data note - we have revised the article in line with your suggestions.

# Reviewer report: Reviewer response for version 1

**DOI:** 10.21956/wellcomeopenres.20755.r62041

**Published:** 2023-08-01

**Authors:** Carel Oosthuizen

**Affiliations:** 1 Zoology and Entomology, University of Pretoria, Pretoria, Gauteng, South Africa

This article reports on the genome assembly of *Sarcophaga subvicina.* Six chromosomes, 5 autosomes and one sex chromosome (X), were defined by assembling the sequence reads.

 I think this is a great report on a study that is well performed with high quality results. The date is well explained and easily accessible in the different forms that it was reported in. Well done to the authors.

 The only suggestion that I would like to make is that there be a paragraph included on the possible reasons why the Y chromosome sequences could not be identified as the authors already know that it should be present.

 Once again, well done.

Are sufficient details of methods and materials provided to allow replication by others?

Yes

Is the rationale for creating the dataset(s) clearly described?

Yes

Are the datasets clearly presented in a useable and accessible format?

Yes

Are the protocols appropriate and is the work technically sound?

Yes

Reviewer Expertise:

Population genetics, epigenetics, population genomics.

I confirm that I have read this submission and believe that I have an appropriate level of expertise to confirm that it is of an acceptable scientific standard.

# Reviewer report: Reviewer response for version 1

**DOI:** 10.21956/wellcomeopenres.20755.r59681

**Published:** 2023-07-05

**Authors:** Paul Mireji

**Affiliations:** 1 Biotechnology Research Institute, Kenya Agricultural and Livestock Research Organization, Kikuyu, Kenya

In this manuscript, the authors isolated nuclear and mitochondrial DNA from single male one male *Sarcophaga subvicina* and sequenced the DNA using Pacific Biosciences  and 10X Genomics platforms, both of which generated  long reads most appropriate for genome assembly due to their better ( 51-65) fold coverage than other technologies such as illimina generated short reads. The assembly was successfully achieved to a high (chromosome) level followed by curation using an appropriate suite of bioinformatic tools and annotations based on transcriptomic data and protein from UniProt database.

 The sample collection, DNA extraction, sequencing and assembly are well described and the results well presented. I however have the following concerns

1. The importance of assembling this genome is not adequately presented, other than as part of a routine process in the as part of the Darwin Tree of Life Project and that that this species is attracted to large carcasses. Is there any other economic, medical/ veterinary etc importance of this species? What knowledge will better be understanding of the advance?
2. While the transcriptomic data was used in the annotation of the genome, information on generation and nature of this data is missing. Was it from the same species? Was it tissue specific or whole body? The nature (source) of this data influences the gene families that will be preferentially annotated. For example, midgut derived RNA seq data will provide better annotation of midgut associated genes such as digestive genes than olfaction genes associated with the antennae.
3. The assembly information would have been improved by information on genome arrangements such as synteny, intergenic sequences, transposon/repetitive sequence expansions, number of exons and their average size and orthologs in relation to closest relative among others.
4. Information on nature of the genes annotated has not been provided. The information would yield important insights with broad implications important aspects of the flesh fly biology. The approach would identify gene families involved in important biological aspects of the fly such as its olfaction, flesh feeding/nutrition, immunity, microbiome, reproduction and developmental biology among other important aspects of this fly

Are sufficient details of methods and materials provided to allow replication by others?

Yes

Is the rationale for creating the dataset(s) clearly described?

Partly

Are the datasets clearly presented in a useable and accessible format?

Yes

Are the protocols appropriate and is the work technically sound?

Yes

Reviewer Expertise:

Medical Entomology, Bioinformatics and Genomics

I confirm that I have read this submission and believe that I have an appropriate level of expertise to confirm that it is of an acceptable scientific standard, however I have significant reservations, as outlined above.

# Response

**Published:** 2024-05-09

**Authors:** Tree of Life Team Sanger

**Affiliations:** Wellcome Trust Sanger Institute, UK

Thank you for your comments on this data note.  We have made a variety of changes in response to reviewers' comments.  In response to points 2, 3 and 4, we would like to point out that we have given links to an external source of annotation of the genome, provided by the European Bioinformatics Institute. We do not provide the annotation, and do not analyse the annotation in this data note. We do not have access to information such as the source of transcriptomic data used, as we have not presented RNA sequencing for this species.
